# Predictors of Recurrence for T3a RCC: A Recurring Conundrum

**DOI:** 10.3390/diagnostics10110983

**Published:** 2020-11-21

**Authors:** Zev Leopold, Arnav Srivastava, Eric A. Singer

**Affiliations:** Section of Urologic Oncology, Rutgers Cancer Institute of New Jersey and Robert Wood Johnson Medical School, New Brunswick, NJ 08902, USA; zrl7@rwjms.rutgers.edu (Z.L.); srivasar@rwjms.rutgers.edu (A.S.)

Although the gold standard treatment for localized renal cell carcinoma (RCC) is radical nephrectomy (RN) or partial nephrectomy (PN), recurrence rates remain high at 7%, 26%, and 39% for T1, T2, and T3 staged disease, respectively [1]. Thus, having a high-fidelity staging system that accurately assesses the risk of recurrence and identifies patients who may benefit from adjuvant therapy and close monitoring is critical to improving oncologic outcomes [2,3,4,5].

In the above article, Shimizu et al. aimed to identify predictors of recurrence after RN or PN in pT3aN0M0 RCC [6]. Current American Joint Committee on Cancer (AJCC) staging distinguishes pT1 and pT2 by size; but both must be confined to the kidney [7]. In contrast, pT3 is not defined by size, but rather by extension into perinephric or sinus fat or involvement of segmental vessels/main renal vein/IVC. As Shimizu et al. point out, this schema causes significant pathological heterogeneity, because tumors of any size with perinephric fat invasion (PFI), renal sinus fat invasion (RSI), and/or renal vein invasion (RVI) are all staged as pT3a [6]. In a study of 91 patients with pT3aN0M0 RCC, the authors sought to identify a correlation between invasion location and recurrence rate. RVI showed a significant difference in recurrence free survival (RFS) on univariate analysis (HR 2.42, 95% CI 1.13–5.21; *p* = 0.022), although this difference was not significant on multivariate analysis (HR 1.73, *p* = 0.39). Neither RSI nor PFI independently or simultaneously showed a significant effect of RFS. Rather, only patients with all three invasion sites—RSI + PFI + RVI—showed reduced a RFS (HR 14.28, 95% CI 3.03–6.71; *p* = 0.0008).

Evidence exploring the prognostic value of invasion location and its effect on recurrence rates has been divergent. Shimizu et al.’s findings are in agreement with some of the previously published literature showing that RSI has no effect on RFS compared to PFI [8,9]. Similarly, although RSI has been associated with a worse five-year disease specific survival (DSS) compared to PFI on univariate analysis, this effect was negated when other tumor characteristics such as Fuhrman grade or tumor diameter were taken into account [10]. Conversely, other studies concluded that RSI is an independent, unfavorable prognostic factor when compared to PFI [11,12]. As such, some conclude that RSI should not only be specified in TNM classification, but that clinical pathological routine should be modified to include sampling of the renal sinus [11,12]. It has been hypothesized that, because the renal capsule terminates in the renal sinus, malignant cells have direct access to an area rich in lymphatics and vasculature allowing for dissemination [6,11]. 

Others argue that RSI and PFI should be excluded from the pT3a classification altogether. Instead, they note that because perinephric fat invasion is determined on post-operative histopathology, it has no bearing on surgical approach. Rather, the pT3 category should only include cases with vascular invasion [13]. However, while Shimizu et al. showed an association between RVI and decreased RFS in univariate analysis (HR 2.42, 95% CI 1.13–5.21; *p* = 0.022), no difference was found in multivariate analysis (HR 1.73, *p* = 0.39). Only invasion of all three sites was significant. Similarly, others have shown that concomitant PFI and RVI represent a particularly poor prognostic factor [14,15]. Interestingly, although RVI alone was not significant, clinically detectable-renal vein thrombosis (cd-RVT) was.

In addition to invasion site, Shimizu et al. explored other prognostic factors of pT3aN0M0 RCC. They found that a size >7 cm had a significant effect on RFS (HR 2.98, 95% CI 1.26–7.05; *p* = 0.013). Others have noted that when compared to pT3a RCC of size ≤ 7 cm, tumors > 7 cm have increased cancer specific mortality (CSM) (HR 1.71, 95% CI 1.31–2.24; *p* < 0.001). An increase in size by 1 cm was associated with a 7% increase in CSM [8]. This has led some to propose a 7 cm cutoff for pT3 RCC to add to the prognostic value of pathologic staging [8,16]. However, it has even been suggested that a 7 cm cutoff for pT3 is only prognostically significant when paired with renal fat invasion [17]. This may be due in part to a correlation between size and Fuhrman grade, with larger tumors more likely to be high grade—a finding also highlighted by Shimizu et al. [6,8,16]. Similarly to RSI and size, urinary collecting system invasion (UCSI) is associated with higher grader, lymph node invasion, and metastasis [18,19]. Shimizu et al. indicate that even when these associations are accounted for on multivariate analysis, UCSI is a poor prognostic factor (HR 4.26, 95% CI 1.86–9.76; *p* = 0.001). Although this echoes some prior literature [20], controversy exists regarding the inclusion of UCSI in T staging [18,19]. 

Overall, it is clear that significant controversy exists regarding the validity of prognostic factors and the appropriate staging of T3 RCC. We commend Shimizu et al. for exploring this essential question and adding their voice to this critical topic. With a sample size of 91 patients and a recurrence rate of 28.6% (n = 26), we encourage additional, larger studies to allow for increased detection. As they note, exploration of prognostic factors is an imperative step in early detection of recurrence, early intervention, and ultimately, improved survival [6]. Furthermore, appropriate staging of malignancies is essential for treatment planning and prognosis [13]. Lastly, the role of neoadjuvant and adjuvant therapy in the treatment of RCC is an area of active investigation [21], and accurate staging is vital for identification of suitable candidates and exploring treatment effectiveness [3].
